# Recurrent Extranodal Diffuse Large B-Cell Lymphoma Manifesting as a Cecal Mass Over 10 Years After Remission

**DOI:** 10.14309/crj.0000000000002075

**Published:** 2026-04-10

**Authors:** Jyoti Yadav, Yeshika Thapa, John Horne, Michelle L. Grant, Danial Nadeem, Kishore Kumar

**Affiliations:** 1 Department of Internal Medicine, The Wright Center for Graduate Medical Education, Scranton, PA; 2 Department of Internal Medicine, UCF/HCA Florida North Florida, Gainesville, Florida, FL; 3 Department of Pathology, Geisinger Community Medical Center, Scranton, PA; 4 Department of Gastroenterology, Geisinger Wyoming Valley, Wilkes-Barre, PA; 5 Department of Gastroenterology, Geisinger Community Medical Center, Geisinger Wyoming Valley, Scranton, PA

**Keywords:** diffuse large B-cell lymphoma, extranodal lymphoma, gastrointestinal lymphoma, urinary obstruction, R-CHOP

## Abstract

Diffuse large B-cell lymphoma (DLBCL) frequently involves extranodal sites, most commonly the gastrointestinal tract; however, predominant colorectal involvement and very late relapse after long-term remission are uncommon. A 70-year-old woman with a history of DLBCL in complete remission for more than 10 years after 6 cycles of R-CHOP presented with progressive weight loss, early satiety, lower abdominal pain, and new-onset urinary urgency and frequency. Cross-sectional imaging revealed a large cecal mass with retroperitoneal extension causing right-sided hydronephrosis. Colonoscopy demonstrated a bulky, ulcerated, partially obstructing mass in the distal ascending colon. Histopathologic evaluation confirmed germinal center B-cell subtype DLBCL with a high proliferation index, and fluorescence in situ hybridization was negative for MYC, BCL2, and BCL6 rearrangements. Bone marrow biopsy showed no evidence of lymphoma, consistent with isolated extranodal relapse. Given frailty, hypoalbuminemia, and prior anthracycline exposure, dose-reduced R-CHOP was initiated with careful limitation of cumulative doxorubicin. This case illustrates a rare presentation of very late, isolated extranodal DLBCL relapse manifesting as a cecal mass with genitourinary obstruction. It underscores the need to consider lymphoma in elderly patients with atypical gastrointestinal and urinary symptoms and highlights the importance of sustained clinical vigilance beyond the conventional 5-year surveillance period in long-term DLBCL survivors.

## INTRODUCTION

Diffuse large B-cell lymphoma (DLBCL) accounts for ∼30%–40% of non-Hodgkin lymphomas and represents a clinically heterogeneous entity with variable patterns of nodal and extranodal involvement. Extranodal disease occurs in up to 40% of cases, with the gastrointestinal (GI) tract being the most frequently affected site; however, primary or predominant colorectal lymphoma remains rare, constituting only a minor proportion of GI lymphomas and colorectal malignancies.^1,2^

Colonic lymphoma often presents with nonspecific symptoms such as abdominal pain, altered bowel habits, anemia, and weight loss, which overlap with presentations of colorectal adenocarcinoma and inflammatory bowel disease and can contribute to diagnostic delay. Most relapses of DLBCL occur within the first 2–3 years after initial therapy, and patients who remain event-free beyond 24–36 months have long-term survival approximating that of the general population, although a persistent, low risk of late relapse beyond 5 years has been documented. This report describes a rare case of very late extranodal GCB-subtype DLBCL relapse presenting as a cecal mass with retroperitoneal extension and ureteral obstruction more than a decade after initial remission.^3^

## CASE REPORT

A 70-year-old woman presented with a 1-week history of intermittent lower abdominal pain, nausea, vomiting, diarrhea, and postprandial fullness, described as a sensation of “a stone stuck in the abdomen.” She reported early satiety for ∼1 month and unintentional weight loss of about 15 pounds over the preceding year. She also endorsed new-onset urinary urgency, frequency, and nocturia without dysuria or gross hematuria.

Her medical history was significant for type 2 diabetes mellitus, hypertension, dyslipidemia, and gout. She was found to have a hypervascular mass involving the left hilum and mediastinum, encasing and compressing the left pulmonary artery, measuring 8.9 × 7.2 × 9.1 cm. Bronchoscopy with biopsy was performed and revealed malignant lymphoma, diffuse type. Based on staging, it was Stage I A. Flow cytometry demonstrated a monoclonal B-cell population with mixed cell size. Immunophenotyping showed positivity for CD45, CD19, CD20, and CD5, with negativity for CD10 and CD23. FMC7 was positive, CD38 showed variable expression (negative/positive), and there was kappa light chain restriction with intermediate intensity. She was previously treated with 6 cycles of R-CHOP between December 2010 and March 2011, after which she achieved complete remission based on clinical assessment and imaging. A computed tomography (CT) scan of the chest performed on August 4, 2011 was unremarkable, and a positron emission tomography (PET) scan dated December 23, 2011 also showed no evidence of disease. Subsequent surveillance imaging, including PET scans, CT chest, and endoscopic evaluation in 2015, were reportedly unremarkable. She denied recent infections, fevers, night sweats, or chills before the presentation.

On examination, she was afebrile and hemodynamically stable. Her body mass index (BMI) was 18 kg/m^2^, reflecting notable weight loss from a BMI of 21.1 (58 kg) in 2023 to 19.0 (46.3 kg) in early 2024. Abdominal examination revealed mild distension and right lower quadrant tenderness without rebound or guarding. No cervical, axillary, or inguinal lymphadenopathy was detected. Cardiopulmonary and neurologic examinations were unremarkable.

Laboratory evaluation showed hyponatremia (sodium 130 mmol/L), mild acute kidney injury with creatinine 1.1 mg/dL (baseline 0.8 mg/dL), elevated lactate of 2.6 mmol/L, leukocytosis with a white blood cell counts of 14,000/μL with neutrophil predominance 87%, and normocytic anemia with hemoglobin 10.3 g/dL. Serum albumin was 2.4 g/dL, consistent with malnutrition. Liver enzymes were mildly elevated (aspartate aminotransferase 37 U/L, alkaline phosphatase 161 U/L) with normal alanine (Placeholder2)aminotransferase. Tumor markers, including carcinoembryonic antigen (1.8 ng/mL) and lactate dehydrogenase (210 U/L), were within reference ranges.

Contrast-enhanced CT of the abdomen and pelvis demonstrated a large, irregular, heterogeneously enhancing cecal mass with contiguous extension into the retroperitoneum. The lesion encased and narrowed the right distal ureter, resulting in right-sided hydronephrosis, and exerted mass effect on both renal veins and the inferior vena cava. There was associated thickening of the ascending colon and a 3.0 × 2.2-cm right lower para-aortic nodal mass. No hepatic or splenic involvement was identified. CT imaging of the neck and chest showed no evidence of cervical, pulmonary, or mediastinal disease. Colonoscopy revealed a large, ulcerated, infiltrative, circumferential lesion in the distal ascending colon causing partial luminal obstruction. The rectum and rectosigmoid colon appeared congested but without discrete masses. Multiple biopsies were obtained from the colonic lesion. A PET–CT scan from the skull base to mid-thigh with fluorodeoxyglucose performed in July 2025 demonstrated no metabolically active lymphoma. The disease was staged as Stage IA.

Histopathologic examination showed diffuse effacement of the colonic architecture by sheets of medium-to-large atypical lymphoid cells with vesicular chromatin and small nucleoli. The tumor cells were strongly positive for CD20 and CD45, expressed Bcl-2, and demonstrated partial Bcl-6 expression, consistent with a germinal center B-cell phenotype. The Ki-67 proliferation index was estimated at 60%–70%. The neoplastic cells were negative for CD10, MUM1, CD30, TdT, cyclin D1, CD23, CD138, EBV-encoded RNA by in situ hybridization, p40, AE1/3, chromogranin, synaptophysin, SOX10, CDX2, CK7, and CK20, effectively excluding carcinoma, neuroendocrine tumor, and mantle cell lymphoma. Fluorescence in situ hybridization showed no rearrangements involving MYC, BCL2, or BCL6. Bone marrow biopsy and flow cytometry demonstrated normocellular marrow without evidence of lymphoma, confirming disease localized to the colon and retroperitoneum.

During hospitalization, the patient developed transient fevers up to 101.5°F and persistent leukocytosis, with a peak white blood cell count of 14.8 K/μL. Blood and urine cultures and a respiratory viral panel were negative, and these abnormalities resolved without targeted antimicrobial therapy. She experienced 6 episodes of hematochezia, with hemoglobin declining from 8.5 g/dL to 7.7 g/dL, attributed to friable lymphoma-infiltrated colonic mucosa and possible chemotherapy-related mucosal injury; no emergent endoscopic or surgical intervention was required.

A left subclavian MediPort was placed, and R-CHOP chemotherapy was reinitiated with a 20% dose reduction due to prior anthracycline exposure, low BMI, and hypoalbuminemia. Prednisone 100 mg orally daily for 5 days was administered with each cycle, and allopurinol was initiated for tumor lysis prophylaxis. Given concerns regarding cumulative doxorubicin-associated cardiotoxicity, a maximum of 2 additional cycles of R-CHOP was planned, with consideration of alternative regimens if further therapy became necessary. Renal function improved with hydration and systemic therapy, and urology recommended close monitoring of hydronephrosis with a low threshold for ureteral stent placement. The patient was discharged in stable condition with coordinated follow-up arranged in hematology-oncology, gastroenterology, and urology clinics.

## DISCUSSION

Extranodal involvement is common in DLBCL, but primary or predominant colorectal presentations remain distinctly uncommon, accounting for only a small fraction of GI lymphomas and colorectal malignancies. Classic criteria for intestinal lymphoma emphasize dominant GI involvement with absent or limited peripheral or mediastinal lymphadenopathy and no early bone marrow or hepatic involvement, a pattern that is compatible with this patient's localized colonic and retroperitoneal relapse.^4^

Clinically, colonic lymphoma often mimics colorectal adenocarcinoma or inflammatory bowel disease, presenting with abdominal pain, altered bowel habits, anemia, and weight loss. In this case, early satiety and postprandial fullness likely reflected partial obstruction and mass effect, whereas urinary urgency and frequency were attributable to retroperitoneal extension with ureteral compression and hydronephrosis. This constellation of symptoms highlights the importance of including lymphoma in the differential diagnosis of right-sided colonic masses, particularly in older patients with a remote history of lymphoma and atypical GI or genitourinary complaints.

**Figure 1.**
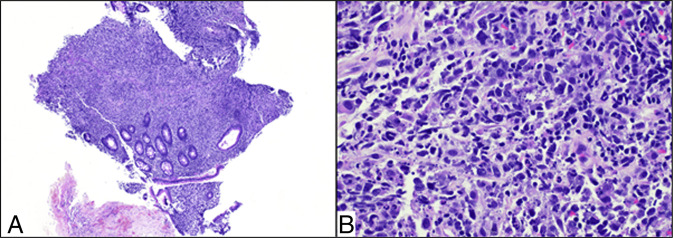
Diffuse large B-cell lymphoma involving colonic tissue; (A) colonic mucosa disrupted by a diffuse aggregate of medium to large atypical lymphoid cells (40×); (B) atypical lymphoid cells displaying variable amounts of fine to vesicular chromatin with multiple prominent nucleoli (400×).

DLBCL is characterized by diffuse infiltration of large, atypical B cells with a high proliferative index, and immunophenotypic and molecular profiles have significant prognostic implications. The immune profile in this case, CD20^+^, CD45^+^, Bcl-2+, partial Bcl-6+, CD10^−^, MUM1−, is consistent with the germinal center B-cell subtype according to Han's algorithm, which in de novo DLBCL is associated with better outcomes compared with the avidin-biotin complex/non-GCB subtype when treated with R-CHOP (Figure 1). The Ki-67 index of 60%–70% is within the range typically seen in aggressive lymphomas and underscores the high proliferative activity of the tumor. Importantly, the absence of MYC, BCL2, and BCL6 rearrangements by fluorescence in situ hybridization effectively excludes double-hit and triple-hit lymphomas, entities that confer particularly poor prognosis and often require more intensive regimens than standard R-CHOP when clinically feasible. Although gene expression profiling and more granular molecular subclassification (including recently defined molecular clusters of DLBCL) further refine risk stratification and may inform targeted approaches, they are not universally accessible and did not alter the core therapeutic paradigm in this older, frail patient.^5–7^ Although molecular subclassification of DLBCL continues to evolve, immunohistochemistry and targeted cytogenetic analysis remain central to routine diagnostic confirmation and therapeutic planning in clinical practice.^8,9^

Management in this case was complicated by advanced age, frailty, hypoalbuminemia, and prior anthracycline exposure. Although standard-dose R-CHOP is the backbone of frontline DLBCL therapy, dose-reduced regimens are commonly employed in older or medically complex patients to balance efficacy and toxicity. The chosen approach dose-reduced R-CHOP with strict limitation of additional doxorubicin reflected an attempt to maintain disease control while mitigating the risk of cardiotoxicity and treatment-related complications. Episodes of hematochezia during therapy were managed conservatively and underscore the need for close multidisciplinary collaboration between gastroenterology, hematology-oncology, and surgery in managing GI complications of both lymphoma and systemic therapy.^10^

Most relapses of DLBCL occur within 2–3 years after diagnosis, and patients who achieve event-free survival at 24–36 months have survival trajectories like the general population. Nevertheless, several cohorts have documented a small but meaningful proportion of late relapses beyond 5 years, with estimates ranging from ∼3% to 10% depending on population and follow-up duration. In contrast, colorectal involvement at the time of relapse is exceptionally rare and has not been specifically reported in the literature. Primary colorectal DLBCL itself accounts for only 0.2%–1.2% of all colorectal malignancies, underscoring the rarity of this site. A very late relapse (>10 years) presenting as isolated primary colorectal DLBCL, therefore, represents a previously undescribed clinical scenario. This case highlights a unique manifestation of late DLBCL relapse and reinforces the importance of long-term surveillance and individualized management strategies in survivors.^11–13^

Current follow-up recommendations generally favor structured clinical assessment every 3-6 months for the first 2 years, then every 6–12 months until 5 years, with subsequent transition to symptom-driven survivorship care rather than routine surveillance imaging in asymptomatic patients. This strategy reflects the high yield of symptom-based detection and the limited utility of routine imaging for identifying relapse in the absence of clinical suspicion.^14^

This case demonstrates that very late relapse, although rare, remains a real possibility and supports sustained clinical vigilance in long-term DLBCL survivors. For clinicians, this translates into (i) maintaining some level of suspicion for relapse in patients with a history of DLBCL who develop unexplained constitutional, GI, or genitourinary symptoms, even beyond a decade after remission; (ii) emphasizing survivorship education so that patients and caregivers recognize and promptly report concerning new symptoms.

Very late recurrence of extranodal GCB-subtype DLBCL presenting as a cecal mass with retroperitoneal extension and ureteral obstruction is rare but clinically significant. This case underscores the importance of considering lymphoma in the differential diagnosis of cecal and ascending colon masses in elderly patients with atypical GI and urinary symptoms and illustrates the complexity of therapeutic decision making in frail individuals with prior anthracycline exposure. It also reinforces the need for guideline-aligned early follow-up and sustained, symptom-directed vigilance throughout long-term survivorship, even beyond the conventional 5-year follow-up period, to facilitate timely recognition and management of late relapse.^12^

## DISCLOSURES

Author contributions: J. Yadav, D. Nadeem, and Y. Thapa wrote and revised the manuscript. K. Kumar overviewed and supervised the manuscript. J. Horne and M. Grant were involved in direct care and provided histopathological reports. J. Yadav is the article guarantor.

Financial disclosure: None to report.

Informed consent was obtained for this case report.
